# IUC24403-78 Relative dose intensity of neoadjuvant chemotherapy predicts pathological response and survival in muscle-invasive urothelial cancer: a multicenter retrospective study

**DOI:** 10.1093/oncolo/oyaf248.021

**Published:** 2025-08-29

**Authors:** G Neola, M Maruzzo, M Maffezzoli, M Rizzo, S Scagliarini, L Danielli, V Conteduca, P Giannatempo, B A Maiorano, L Formisano

**Affiliations:** Department of Medicine and Surgery, Federico II University, Naples, Italy; Oncology Unit 3, Istituto Oncologico Veneto IOV—IRCCS, Padova, Italy; Portsmouth Hospitals University NHS Trust, Portsmouth, United Kingdom; Medical Oncology Unit, Azienda Ospedaliero Universitaria Consorziale Policlinico di Bari, Bari, Italy; UOC di Oncologia, Azienda Ospedaliera di Rilievo Nazionale Cardarelli di Napoli, Naples, Italy; Department of Medical and Surgical Sciences DIMEC, University of Bologna, Bologna, Italy; Unit of Medical Oncology and Biomolecular Therapy, Department of Medical and Surgical Sciences, University of Foggia, Policlinico Riuniti, Foggia, Italy; Medical Oncology Unit, Fondazione IRCCS Istituto Nazionale dei Tumori, Milan, Italy; Department of Medical Oncology, IRCCS San Raffaele Hospital, Milan, Italy; Department of Medicine and Surgery, Federico II University, Naples, Italy

## Abstract

**Background:**

The relative dose intensity (RDI) of neoadjuvant chemotherapy (NAC) is known to influence pathological response and survival in several solid tumors. However, its role in muscle-invasive urothelial carcinoma (MIUC) remains underexplored. This study aimed to evaluate the association between RDI of cisplatin-based (CB) NAC and pathological complete response (pCR), and its correlation with event-free survival (EFS) and overall survival (OS).

**Methods:**

We retrospectively collected data on 301 patients with cT2-cT4 ± N0-N+ MIUC of CB NAC followed by radical cystectomy across multiple centers in Italy and the UK between 2014 and 2025. RDI was defined as the ratio between the dose of chemotherapy administered and the planned dose, multiplied by the ratio between the planned duration and the actual duration of treatment, expressed as a percentage. The planned dose was 80 mg/m^2^ of cisplatin per cycle over 4 cycles within 63 days. The primary endpoint was pCR (ypT0/ypTis); secondary endpoints included EFS and OS. To limit heterogeneity in drug delivery, only patients who received 3 or 4 cycles of NAC were included. Associations were analyzed through χ^2^ tests and Kaplan–Meier/log-rank methods. A multivariate logistic regression was performed, including RDI, cT stage, nodal status, Charlson Comorbidity Index, age, sex, performance status, nephrostomy, and histology.

**Results:**

Among 306 patients, 161 were included in the RDI-High and 145 in the RDI-Low group. Median follow-up time was 32.7 months (95%CI: 29.9-35.6). pCR rate was significantly higher in the RDI-High than in the RDI-Low group (47.8% vs 31.7%, *P* = .004) (Figure 1).

**Conclusions:**

Maintaining an RDI ≥80% is significantly associated with higher pCR rates and correlates with improved EFS and OS. These findings underscore the importance of preserving a high NAC RDI value in MIUC.

**Figure 1.**
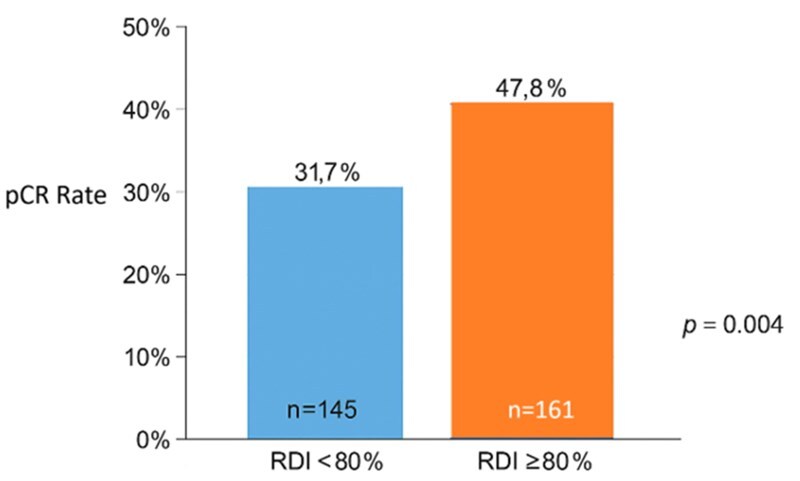
pCR Rate according to RDI values. In multivariate analysis, RDI ≥80% remained an independent predictor of pCR (OR = 2.78, 95%CI: 1.55-5.01; *P* < .001). RDI ≥80% showed a favorable trend toward improved EFS (HR = 0.69, 95%CI: 0.46-1.04; *P* = .080) and OS (HR = 0.61, 95%CI: 0.35-1.06; *P* = .083). Achieving pCR was strongly associated with reduced risk of recurrence (EFS HR = 0.18, 95%CI: 0.10-0.31; *P* < .001) and death (OS HR = 0.28, 95%CI: 0.14-0.54; *P* < .001).

